# Filamentation of hCTPS1 with CTP

**DOI:** 10.1186/s13578-025-01450-6

**Published:** 2025-07-30

**Authors:** Chen-Jun Guo, Xiaojie Bao, Ji-Long Liu

**Affiliations:** 1 School of Life Science and Technology, ShanghaiTech University, Shanghai, China; 2 Department of Physiology, Anatomy and Genetics, University of Oxford, Oxford, UK

**Keywords:** CTP synthase, Cytoophidium, Metabolic filament, Cryo-EM, Product feedback regulation

## Abstract

CTP synthase (CTPS) is a key enzyme in *de novo* CTP synthesis, playing a critical role in nucleotide metabolism and cellular proliferation. Human CTPS1 (hCTPS1), one of the two CTPS isoforms, is essential for immune responses and is highly expressed in proliferating cells, making it a promising therapeutic target for immune-related diseases and cancer. Despite its importance, the regulatory mechanisms governing hCTPS1 activity remain poorly understood. Here, we reveal that CTP, the product of CTPS, acts as a key regulator for hCTPS1 filamentation. Using cryo-electron microscopy (cryo-EM), we resolve the high-resolution structure of CTP-bound hCTPS1 filaments, uncovering the molecular details of CTP binding and its role in filament assembly. Importantly, we demonstrate that CTP generated from the enzymatic reaction does not trigger filament disassembly, suggesting a conserved regulatory pattern. Furthermore, by analyzing the binding modes of two distinct CTP-binding pockets, we provide evidence that this filamentation mechanism is evolutionarily conserved across species, particularly in eukaryotic CTPS. Our findings not only elucidate a novel regulatory mechanism of hCTPS1 activity but also deepen the understanding of how metabolic enzymes utilize filamentation as a conserved strategy for functional regulation. This study opens new avenues for targeting hCTPS1 in therapeutic interventions.

**Supplementary Information:**

The online version contains supplementary material available at 10.1186/s13578-025-01450-6.

## Introduction

Cytidine triphosphate synthase (CTPS) is a pivotal metabolic enzyme that sits at the crossroads of nucleotide biosynthesis, playing a central role in the synthesis of DNA, RNA, and phospholipids [1–5]. As the rate-limiting enzyme in the *de novo* CTP synthesis pathway, CTPS not only drives cellular proliferation but also tightly regulates the intracellular CTP pool, making it essential for maintaining metabolic homeostasis [6–10].

Structurally, CTPS functions as a homotetramer, with each monomer comprising an N-terminal amidoligase (AL) domain and a glutamine amidotransferase (GAT) domain [11, 12]. The enzyme catalyzes a multi-step reaction: the AL domain phosphorylates uridine triphosphate (UTP) using ATP, generating an unstable intermediate, 4-phospho-UTP (4-Pi-UTP) [13, 14]. This intermediate is then aminated by ammonia, which is hydrolyzed from glutamine in the GAT domain and transported through an intramolecular ammonia tunnel [14, 15]. The reaction is finely tuned by allosteric regulators such as GTP, which stabilizes intermediate states, and feedback-inhibited by its end product, CTP [14, 16–19]. These regulatory mechanisms, along with the tetrameric structure, are highly conserved across species, underscoring the fundamental role of CTPS in cellular metabolism [11, 20–23].

Beyond its enzymatic activity, CTPS exhibits a remarkable ability to polymerize into large-scale filamentous structures known as cytoophidia [24]. These structures have been observed in diverse organisms, including bacteria, yeast, fruit flies, and human cells, suggesting an evolutionarily conserved regulatory mechanism [24–28]. Filamentation is thought to modulate CTPS activity, although the precise mechanisms remain elusive [14, 24–30].

In several mammals, including humans, CTPS exists as two isoforms—CTPS1 and CTPS2—that share 75% sequence identity but distinct physiological roles [7]. Human CTPS1 (hCTPS1) is widely expressed in proliferating cells and is critical for immune function, as its deficiency leads to severe immune impairment [6, 7]. Notably, CTPS1 is overexpressed in many cancer cells, and its activity is essential for the replication of viruses, pathogens, and parasites, making it a promising therapeutic target for cancer, infectious diseases, and immune disorders [4, 31–39]. Despite its biomedical significance, the regulatory mechanisms governing hCTPS1 activity, particularly its filamentation dynamics, remain poorly understood.

Previous studies on eukaryotic CTPS, including *Drosophila* CTPS (DmCTPS) and hCTPS2, have revealed that filamentous structures adopt two distinct conformations, enabling enzymatic regulation through ligand binding and release [23, 29]. For hCTPS1, it has been proposed that filament formation is induced by substrate binding, while product (CTP) binding triggers filament disassembly [30]. However, due to limitations in structural resolution and experimental conditions, the molecular details of how CTP binding regulates hCTPS1 filamentation and enzymatic activity remain unclear.

In this study, we address this critical gap by investigating the impact of CTP on hCTPS1 filamentation. Using cryo-electron microscopy (cryo-EM), we resolve the high-resolution (3.3 Å) structure of hCTPS1 filaments bound to CTP, revealing the precise binding modes and conformational changes associated with filament assembly. Our findings demonstrate that CTP, the enzymatic product, does not induce filament disassembly, challenging the prevailing model of hCTPS1 regulation. Furthermore, we provide evidence that this filamentation mechanism is evolutionarily conserved, particularly in eukaryotic CTPS. These insights not only elucidate a novel regulatory mechanism of hCTPS1 but also deepen our understanding of how metabolic enzymes utilize filamentation as a conserved strategy for functional regulation. This work opens new avenues for targeting hCTPS1 in therapeutic interventions for cancer, immune disorders, and infectious diseases.

## Results

### hCTPS1 assembles into large filamentous structures

**Fig. 1:**
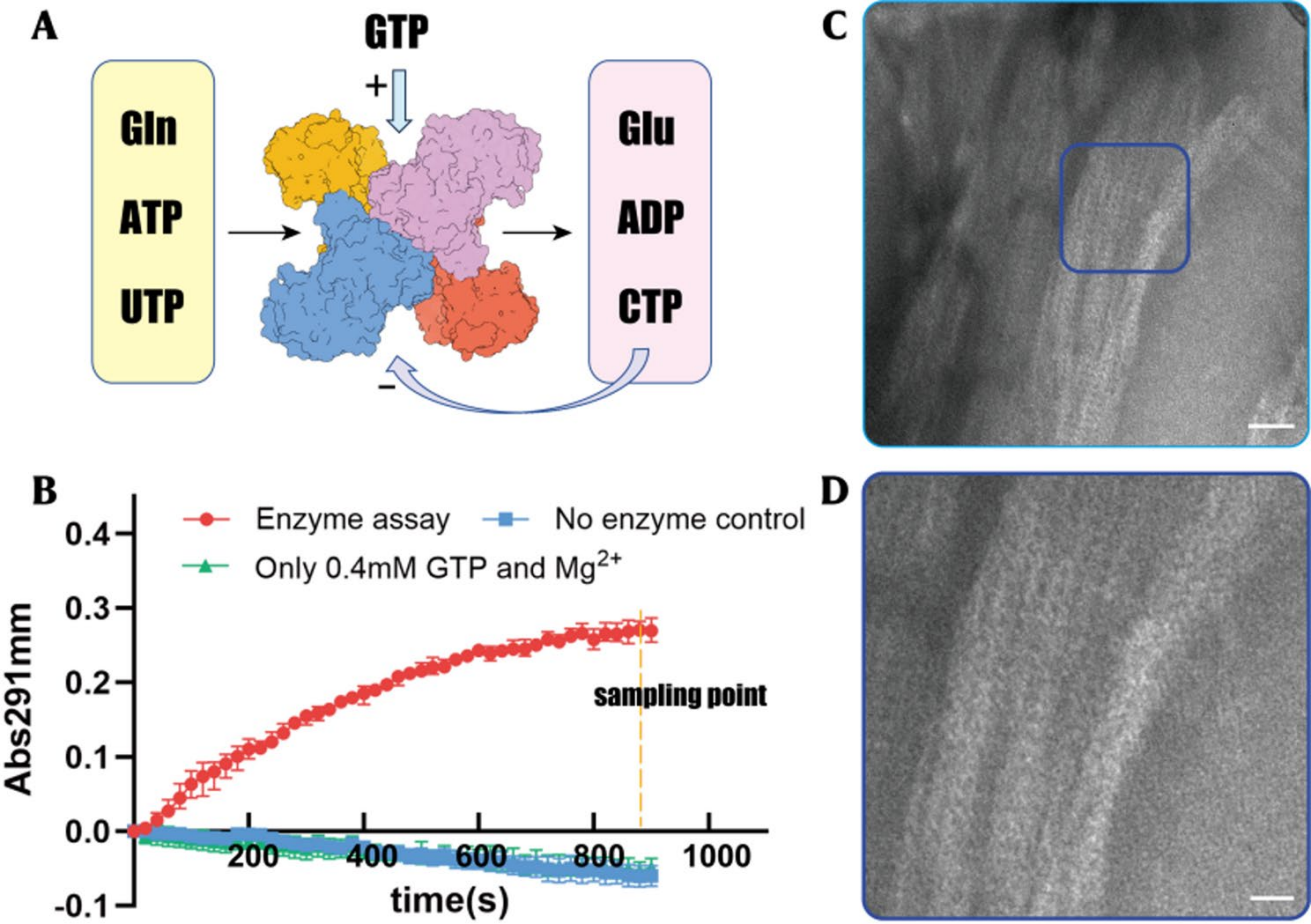
Catalytic mechanism and enzymatic reaction of hCTPS1. (**A**) Reaction scheme of CTP synthase. UTP accepts a phosphoryl group hydrolyzed by ATP and form an intermediate 4-Pi-UTP in the GAT domain (grey box). The ammonia is released from glutamine in the AL domain (yellow box) and then ligated to 4-Pi-UTP to form CTP in GAT domain. (**B**) Kinetic curves of hCTPS1 enzyme assay in 0.4 mM GTP and control experiments. The X-axis indicates time. The Y-axis indicates the change in absorbance 291 nm. (**C**-**D**) Negative-stain EM micrographs of hCTPS1 at the end of enzymatic reaction. Scale bar, 50 nm. (**D**) is the zoomed-in view of the blue box in (**C**). The scale bar is 200 nm

hCTPS1 functions as a homotetramer, with each monomer comprising a glutamine amidotransferase (GAT) domain and an amidoligase (AL) domain. In the presence of GTP, the GAT domain hydrolyzes glutamine (Gln) to produce ammonia, which is transferred to the AL domain to catalyze the conversion of uridine triphosphate (UTP) to cytidine triphosphate (CTP) via an ATP-dependent intermediate, 4-phospho-UTP (4-Pi-UTP) (Fig. 1A). Purified hCTPS1 exhibited high homogeneity and robust catalytic activity in enzyme assays (Fig. 1B).

In the enzymatic reaction system containing magnesium ions, GTP, and excess Gln, the reaction produced ADP, glutamate (Glu), and CTP. After 1200 s of reaction with 1 mM UTP and 1 mM ATP, the system generated approximately 0.45 mM CTP, achieving a conversion of 45% and a final UTP:CTP ratio of 11:9. Strikingly, cryo-electron microscopy analysis of the reaction solution revealed large, stacked bundles composed of periodically arranged filamentous structures (Fig. 1C, D). Grayscale analysis confirmed the uniformity of these structures, suggesting that hCTPS1 can form filaments and further aggregate into higher-order bundles in CTP-rich environments.

### CTP induces hCTPS1 filamentation

**Fig. 2:**
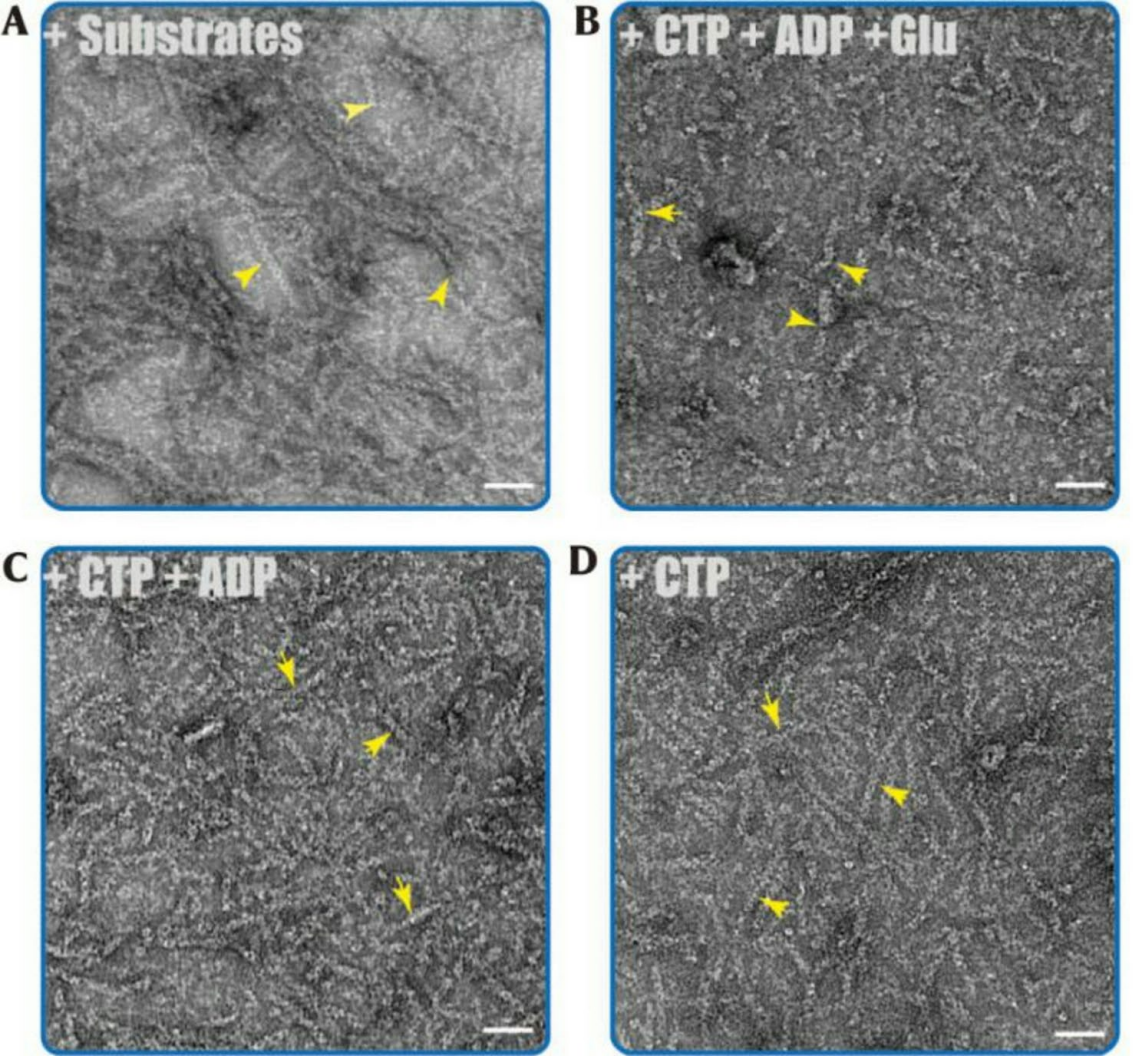
Negative staining of hCTPS1 filament. The wild-type hCTPS1 can form filament under several condition with Mg^2+^. Scale bar, 50 nm. Filaments are indicated by yellow arrows. (**A**) Negative-staining EM micrographs of hCTPS1 incubated with all substrates (ATP, UTP, GTP and Gln) combinations. (**B**) Negative staining of hCTPS1 incubated under all product ligands condition. (**C**) hCTPS1 forms filaments when incubated with CTP and ADP. (**D**) Incubation of hCTPS1 with CTP alone is sufficient to induce filament polymerization.

To investigate the mechanism underlying hCTPS1 filamentation, we explored the effects of various ligands on its self-assembly. Previous studies have shown that hCTPS1 forms filaments in the presence of substrates (ATP and UTP) and the allosteric regulator GTP (Fig. 2A). Here, we demonstrated that hCTPS1 also forms filaments in solutions containing the enzymatic products CTP, ADP, and Glu (Fig. 2B), indicating that product binding can induce filament assembly.

By systematically reducing the types of ligands in the reaction system, we identified CTP and ADP as key inducers of hCTPS1 filamentation. Remarkably, hCTPS1 formed filaments in the presence of CTP alone (Fig. 2C) or ADP alone, but not in systems containing only Glu. These results suggest that CTP, this end product, can coexist with hCTPS1 filaments, challenging the prevailing view that CTP triggers filament disassembly.

To further validate this finding, we conducted a time-point sampling in which CTP was added to hCTPS1 and immediately imaged using negative-staining electron microscopy. Filamentous structures were observed within seconds, confirming that CTP rapidly induces hCTPS1 filamentation (Figure S1). This finding contradicts previous models and suggests that CTP-mediated feedback inhibition of hCTPS1 does not involve filament disassembly.

### Cryo-EM structure of CTP-bound hCTPS1 filament

To elucidate the structural basis of hCTPS1 filamentation under product-bound conditions, we resolved the cryo-EM structure of hCTPS1 filaments in the presence of CTP (Figure S2&S3). To stabilize the GAT domain, we included 6-diazo-5-oxo-L-norleucine (DON), a glutamine analog that irreversibly inhibits CTPS activity by covalently binding to the glutamine site. Negative-staining experiments confirmed that DON does not disrupt filament formation.

**Fig. 3:**
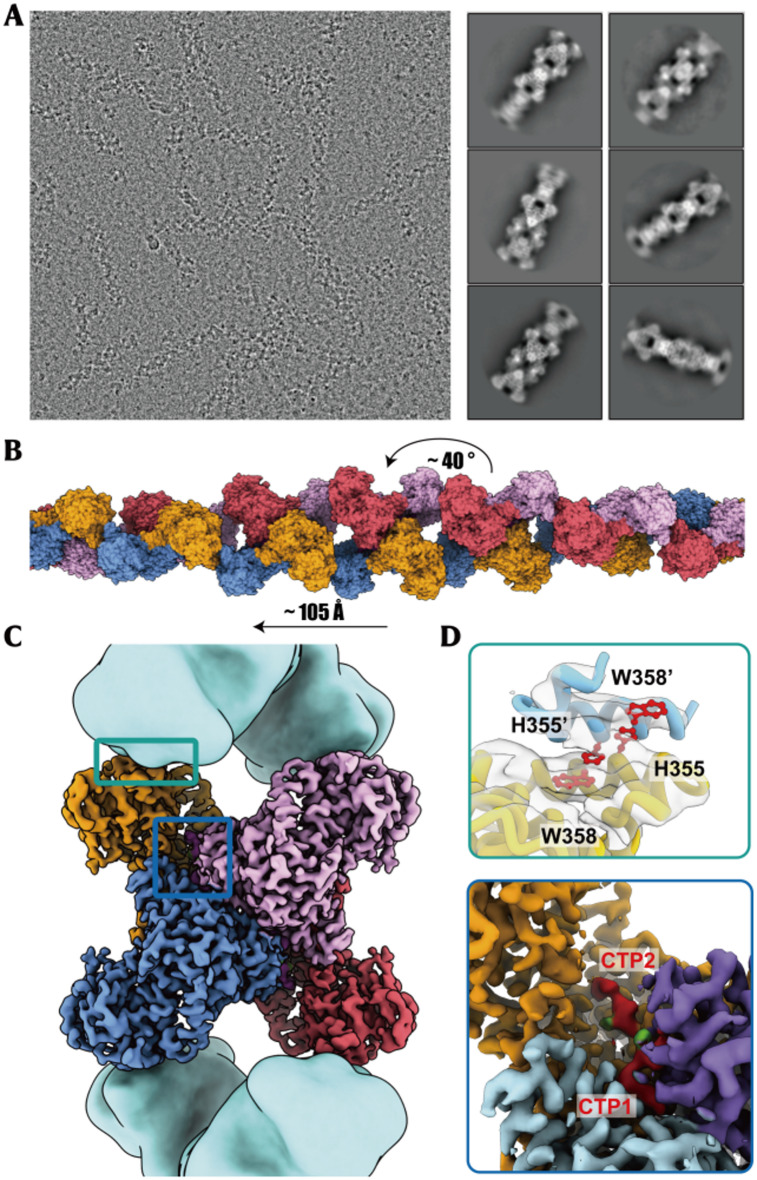
Cryo-electron microscopy (cryo-EM) analysis and overall structure of hCTPS1 filaments with CTP. (**A**) Cryo-electron micrograph of hCTPS1 under CTP and DON condition. Several representative 2D class averages of the hCTPS1 filament are selected in view of the RELION-4 on the right. (**B**) Schematic model of hCTPS1 filament. CTP bound filament model twists 40° between two adjacent tetramers and shifts along the axis 105 Å (**C**) Cryo-EM structure of hCTPS1 bound to CTP and DON. The central hCTPS1 tetramer is colored by protomer. (**D**) Zoomed-in view of the top green box in (**C**), showing the interface of two adjacent tetremers. The density of residues responsible H355 and W358 for the interactions are color red. Zoomed-in view of the below blue box in (**C**), showing the CTP in the non-canonical site.

Cryo-EM analysis revealed clear filamentous structures, and 2D classification highlighted detailed structural features (Fig. 3A). Using 312,573 particles for reconstruction, we achieved a final resolution of 3.3 Å. The structure showed that hCTPS1 filaments consist of helical arrays of tetramers, with each helical unit rising by 105 Å and twisting by approximately 40° (Fig. 3B, C). The tetramers are connected through α-helix-mediated interactions involving residues 346–357 in the GAT domain. Notably, residues H355 and W358, which are critical for filament assembly across species, exhibited continuous electron density in the density map, underscoring their conserved role (Fig. 3D). Each tetramer contained two distinct CTP binding sites, providing insights into the molecular basis of CTP-mediated regulation.

### Conserved CTP binding modes in hCTPS1 filaments

**Fig. 4:**
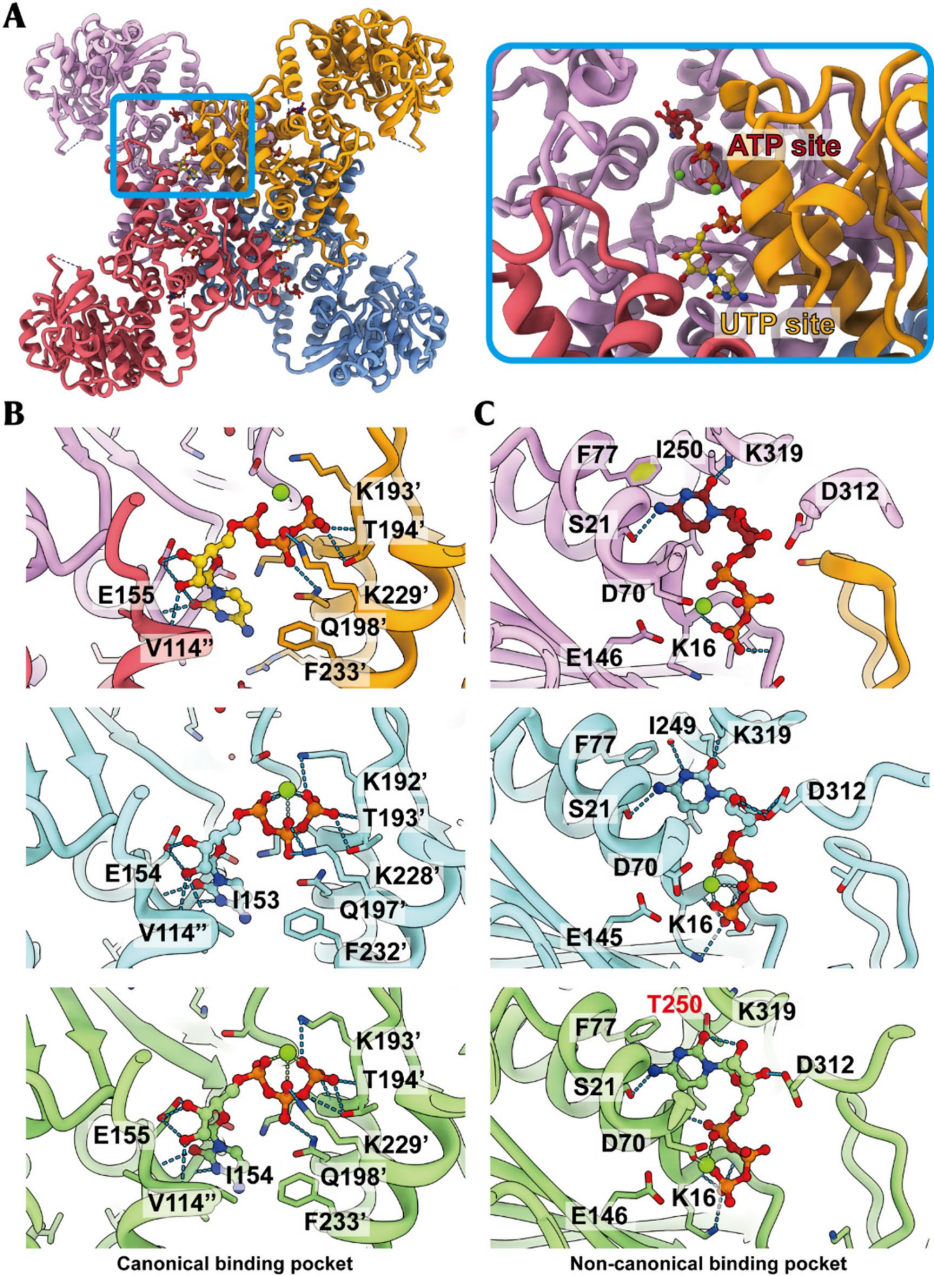
CTP binding modes in hCTPS1 and comparison of CTP binding pockets among organisms. (**A**) A single refined hCTPS1 tetramer model from the filament, colored by protomer (pink, goden, blue and red) and nucleotide highlighted in yellow and crimson. Zoomed-in view of the below blue box shows the CTP in the canonical(yellow) and non-canonical(crimson) site, which are respectively occupied by UTP and ATP under substrate state. (**B**-**C**) Diversities of CTP binding pockets among organisms. Models of hCTPS1, *Drosophila* cytidine triphosphate synthase(DmCTPS) and hCTPS2 are colored in pink, blue, and green, respectively, with the corresponding Protein Data Bank (PDB) codes 9vmm, 7dpw, and 7mh1. (**B**) Canonical CTP binding pocket. CTP is represented by yellow sphere-and-stick models, hydrogen bonds are shown as blue dashed lines, and magnesium ions(Mg^2^^+^) represented by green spheres. (**C**) Comparisons of non-canonical CTP binding sites

CTPS is known to undergo product feedback inhibition, where high CTP concentrations induce conformational changes that reduce enzymatic activity. In our structure, two CTP molecules were observed bound to each protomer: one at the interface of three protomers (overlapping with the UTP binding site) and the other within a single protomer (overlapping with the ATP binding site) (Fig. 4A). We designated these as the canonical and non-canonical binding pockets, respectively.

In the canonical binding pocket, the triphosphate group of CTP is stabilized by electrostatic and hydrogen-bonding interactions with each protomer. Residue E155 forms salt bridges with the ribose group, while the cytosine base interacts electrostatically with the backbone of residue E155. Sequence alignment revealed that the residues involved in CTP recognition in this pocket are highly conserved in hCTPS2 and *Drosophila* CTPS (DmCTPS) (Fig. 4B & Figure S5, 6).

In the non-canonical binding pocket, the triphosphate group interacts electrostatically with a loop, while the cytosine group is stabilized by interactions with S21, K319, and F77. These residues are identical in DmCTPS and highly conserved in hCTPS2, except for T250, which differs between hCTPS1 (I250) and hCTPS2 (T250). This difference has been exploited to design selective inhibitors targeting hCTPS1 (Fig. 4C) [40].

### Conserved conformational changes in hCTPS1 filaments

**Fig. 5:**
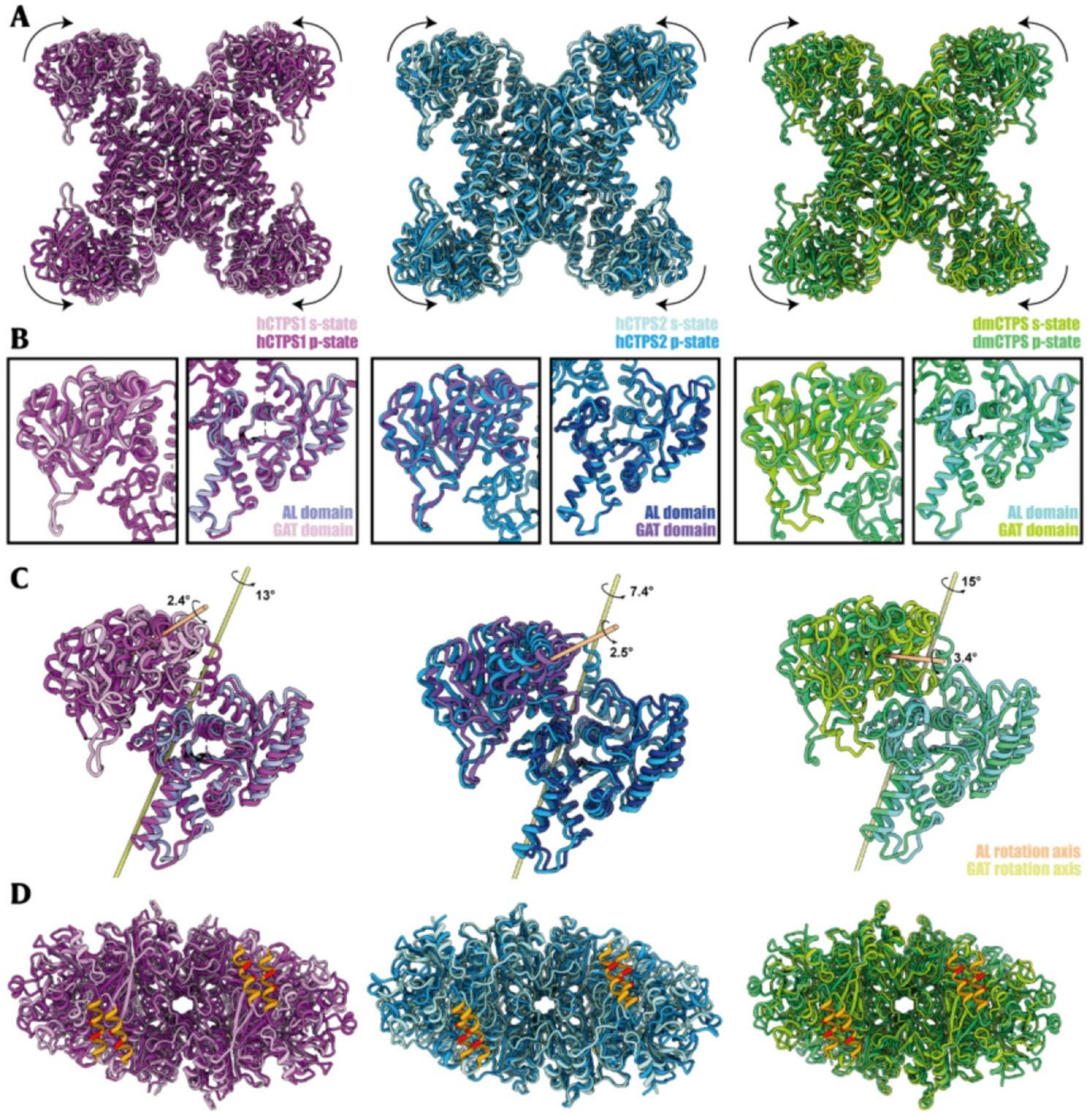
Structural comparison between hCTPS1(purple), hCTPS2(blue) and DmCTPS(green) in substrate-state and product-state. The structures of hCTPS1, hCTPS2 and DmCTPS in s-state are colored in pink, powder blue and lime green, with the corresponding Protein Data Bank (PDB) codes 7mgz, 6pk4, and 7dpt. The structural comparison of hCTPS1, hCTPS2 and DmCTPS in p-state are colored in purple, deep sky blue and forest green, with the corresponding Protein Data Bank (PDB) codes 9vmm, 7mh1, and 7dpw. (**A**) Structural comparison between hCTPS1 tetramer, hCTPS2 tetramer and DmCTPS tetramer in substrate-state and product-state. (**B**) Structural comparison of AL domain and GAT domain. The wing structure in GAT domain has deviated among the three species, while the conformation of AL domain remains almost unchanged. (**C**) Rotation axis of AL domain and GAT domain transitioning between two states. (**D**) Comparison of the interaction interface between adjacent tetramers in filament. H355 and W358 are colored red

Like hCTPS2 and DmCTPS, hCTPS1 filaments exhibit distinct conformations under substrate-bound and product-bound conditions. While the filament assembly interface remains unchanged, the helical parameters of the filaments vary with ligand binding (Fig. 5A & Figure S7).

Structural alignment of the AL domain (residues 1–270) between substrate-bound (s-state) and product-bound (p-state) hCTPS1 revealed an RMSD of 1.267 Å, with conformational differences localized to the Arch region (residues 45–67) and Loop183–194. The GAT domain exhibited high rigidity, with an RMSD of 0.899 Å for all 249 Cα atoms, indicating stable conformations under different ligand conditions (Fig. 5B).

Further analysis showed that the conformational changes in the hCTPS1 tetramer primarily involve rotational displacement of the GAT domain, while the AL domain remains relatively fixed (Fig. 5C). The filament interface on the GAT domain surface maintains a stable, outward-facing conformation, enabling antiparallel assembly of tetramers. This assembly mode is conserved in hCTPS2 and DmCTPS (Fig. 5D).

In summary, hCTPS1 filaments exhibit conserved conformational changes and assembly interfaces under substrate and product conditions, highlighting the evolutionary conservation of the filamentation regulatory pattern.

## Discussion

### CTP promotes hCTPS1 filamentation

Previous studies suggested that hCTPS1 filaments are stable only in the presence of substrates, and that binding to the product CTP triggers filament disassembly, leading to negative feedback inhibition [30]. However, our findings challenge this model by demonstrating that hCTPS1 can form stable filaments in the presence of CTP. We resolved the high-resolution structure of CTP-bound hCTPS1 filaments, revealing that CTP generated during enzymatic reactions does not induce filament depolymerization. This discovery provides a new perspective on the regulatory mechanisms of hCTPS1 activity.

The two CTP binding pockets in hCTPS1 exhibit ligand recognition modes highly similar to those of *Drosophila* CTPS (DmCTPS) and hCTPS2 [14, 29]. The canonical binding pocket, located at the interface of three monomers, overlaps with the UTP binding site, while the non-canonical binding pocket occupies the ATP/ADP binding site. Although the competitive binding mechanism at the non-canonical site remains to be fully elucidated, our structural data show that the phosphate backbone of CTP shares binding pockets with UTP and ATP, highlighting a conserved mechanism of ligand recognition and stabilization across species.

Notably, the formation of hCTPS1 filaments in the presence of CTP is rapid and robust. Filament assembly occurs almost immediately after incubation, and prolonged incubation leads to the formation of large, stable bundles. This suggests that the filamentous structure of hCTPS1 bound to CTP is highly stable, further supporting the idea that CTP-mediated regulation does not involve filament disassembly.

### Filamentation of hCTPS1 and hCTPS2

hCTPS1 and hCTPS2 play critical roles in cell proliferation and immune function, yet their precise molecular mechanisms remain poorly understood [6, 7]. hCTPS1, in particular, is essential for the proliferation of cancer cells, such as Jurkat cells, and its inhibition is considered a promising therapeutic strategy for cancer treatment [4, 7, 31–33, 37–39]. Additionally, hCTPS1 is implicated in severe human diseases and post-transplant medication, making it a valuable drug target. A detailed understanding of its ligand binding and filamentation mechanisms is crucial for developing hCTPS1-specific inhibitors.

Our study reveals that the filament interfaces of hCTPS1, hCTPS2, and DmCTPS are highly conserved, with interactions mediated by an α-helix (residues 346–357). These interfaces differ from those of *Escherichia coli* CTPS (EcCTPS), highlighting evolutionary divergence between prokaryotic and eukaryotic CTPS. Despite these differences, the rotational displacement of the GAT and AL domains during ligand binding and release is conserved, suggesting a shared regulatory mechanism.

This study addresses a critical gap in our understanding of hCTPS1 filamentation. While the conformational changes observed in CTP-bound filaments do not fully explain the transition between filaments and tetramers, they provide valuable insights into the regulation of hCTPS1 activity. The discovery of the CTP-bound hCTPS1 filament structure offers a reliable foundation for the development of targeted therapies.

### CTPS filamentation in eukaryotes and prokaryotes

Filamentation is a conserved regulatory mechanism among metabolic enzymes, influenced by ligand binding, nutrient availability, and pH. In eukaryotes, DmCTPS and hCTPS2 can form filaments under both substrate-bound and product-bound conditions, with substrate-bound filaments exhibiting enhanced enzymatic activity. In contrast, EcCTPS forms filaments only in the presence of products, which inhibit enzyme activity.

We propose that metabolic enzymes, including hCTPS1, regulate complex physiological functions through conformational changes between free polymers and filaments. These structural changes enable efficient ligand binding and release, facilitating enzymatic reactions and maintaining dynamic equilibrium. In vivo, hCTPS likely assembles from monomers into tetramers, which then form filaments and bundle into cytoophidia. These structures persist throughout the cell cycle, rapidly regulating enzyme activity and other biochemical functions.

There is high sequence identity (~ 75%) between hCTPS1 and hCTPS2, and their filament assembly and interfaces are highly conserved, with only minor differences in helical parameters. The relative independence of the GAT and AL domains during conformational changes suggests that hCTPS filaments in vivo may not consist solely of homologous monomers (Figure S8). Future studies should explore the assembly mechanisms and functions of hCTPS filaments and cytoophidia in their native cellular context.

## Methods

### Purification of hCTPS1

hCTPS1 was expressed in Saccharomyces cerevisiae strain BY4741, as given by Chia-Chun Chang and Yi-Lan Li of Liu Lab, which directs expression of C-terminal 6× His-tagged hCTPS1 from the GAL1 promoter. BY4741 were grown in 1× YPD media and induced in 1× YPG media at 30℃. Cells were collected and pelleted by centrifugation at 4,000 rpm for 10 min at 4 ℃. Cell pellets were resuspended in lysis buffer (500 mM NaCl, 50 mM Tris-HCl, 10% glycerol, 1mM PMSF, 5mM β-mercaptoethanol, 5mM benzamidine, 1 µg/mL leupeptin, pH8.0). Lysates were clarified by centrifugation at 10,000 g for 45 min at 4 ℃ in a Beckman Avanti JXN-26 JA-25.50 rotor. Supernatant was incubated with equilibrated Ni-Agarose (Qiagen, Germany) for 1 h. The column was eluted with lysis buffer with 250 mM imidazole and 1 mM β-Me at pH8.0. Elution was concentrated, flash-frozen in liquid nitrogen and stored at -80℃.

### Negative-stain electron microscopy

5 µM hCTPS1 protein with diverse ligands was dissolved in a buffer containing 25 mM Tris HCl, 150 mM NaCl, pH8.0 and 10 mM MgCl2, 10 mM DTT to form filaments. Samples for negative-stain EM were prepared by applying 5.8 µl hCTPS1 to hydrophilic carbon-coated grids (400mech, Zhongjingkeyi Technology Co., China) and staining with 0.1% uranyl formate. Solution systems were incubated alternately at 23.5 ℃ and 0 ℃, with each temperature sustaining 2–5 min and the total duration within 10 min, or incubated at 23.5 ℃ for 15–20 min continuously. Imaging was on a 120 kV microscope (Talos L120C, ThermoFisher, USA) with an Eagle 4 K× 4 K CCD camera system (Ceta CMOS, ThermoFisher, USA) and images were acquired at 57,000× magnification.

### Cryo-EM sample preparation and data collection

To prepare samples for cryo-EM, hCTPS1 was applied to glow-discharge amorphous alloy film (No. M01-Au300-R1.2/1.3) and blotted 2.7–3 µl, then plunged into liquid ethane using a FEI Vitrobot (4℃ temperature, 3–3.5 s blotting time, -1 to 1 blot force). For preparing the CTP-binding hCTPS1 sample, 5 µM hCTPS1 protein was incubated in the buffer with 2 mM CTP, 0.6 µM DON, 25 mM Tris HCl and 150 mM NaCl. Solution systems were incubated alternately at 23.5 ℃ and 0 ℃ for 10 min, each temperature sustaining 2–5 min, or at 23.5 ℃ for 15–20 min continuously. Each sample was diluted twice before boltting. Images were taken with a Gatan K3 summit camera on a FEI Titan Krios electron microscope operated at 300 kV. The magnification was 29,000× in super-resolution mode with the defocus rage − 1.5 to − 2.5 μm and a pixel size of 0.41 Å. The total dose was 60 electrons/Å^2^ subdivided into 40 frames at 2.4-s exposure using SerialEM.

### Cryo-EM data processing

The whole workflow was performed in RELION GUI. Movies were dose-weighted, aligned and summed by MotionCor2 through Relion. CTF parameters were assessed by CTFFIND4. Respectively, 95,054 particles were automatically picked and performed 2-dimensional (2D) classification. After 2-dimensional and 3-dimensional (3D) classification, 62,319 particles were selected for the 3D refinement to generate two maps of 3.5Å. CTF refinement and Bayesian polishing were applied to each particle. Finally, we constructed maps with resolution 3.3 Å.

### Model building and refinement

The human CTPS1 monomer model from AlphaFold was applied for the initial models. The tetramer models were generated and docked into the electron density map by Chimera v.1.14. Manual adjustment and rebuilding were performed with Web-coot [41]. Phenix was used to achieve real space refinements. All figures were generated using UCSF Chimera, ChimeraX and Phenix [42, 43]. The datas of helical parameters were estimated by cryosparc helical refinement.

### hCTPS1 enzyme assay

8µM purified hCTPS1 at concentrations as described in each experiment was incubated in the buffer containing 150mM NaCl, 25mM Tris-HCl pH 8.0, 1 mM ATP, 1 mM UTP, 0.4 mM GTP, and 10 mM MgCl_2_ for 20 min at 37℃. 10mM Gln was added to the mixture after prewarmed as initiator. The components of the no enzyme control included 150mM NaCl, 25mM Tris‐HCl pH 8.0, 1 mM ATP, 1 mM UTP, 0.4 mM GTP, 10mM Gln and 10 mM MgCl_2_. The components of the GTP and Mg^2+^ control only included 150mM NaCl, 25mM Tris‐HCl pH 8.0, 0.4 mM GTP and 10 mM MgCl_2_. Absorbance at 291 nm of each mixture(150ul) was measure using SpectraMax i3 to indicate the production of CTP, and measurements were made at 20s intervals for at less 30 min until the absorption value tended to be stable [44].

## Electronic supplementary material

Below is the link to the electronic supplementary material.

Supplementary Material 1

Supplementary Material 2

## Data Availability

The structure data accession codes are EMD-65191 and PDB-9vmm.
