# Supplementary material for: Filamentation of hCTPS1 with CTP
Source: Cell Biosci. 2025 Jul 30;15:112. doi: 10.1186/s13578-025-01450-6 (PMC12312343; doi:10.1186/s13578-025-01450-6)
Supplement: Supplementary file 1 — Supplementary Material 1 [file 13578_2025_1450_MOESM1_ESM.docx]

**Table S1. Cryo-EM data collection and model refinement**

|  |  |
| --- | --- |
| Data collection |  |
| EM equipment | Titan Krios |
| Detector | K3 camera |
| Magnification | 29,000× |
| Voltage (kV) | 300 |
| Electron exposure ((e–/Å2)) | 60 |
| Defocus range(μm) | -1.5 to -2.5 |
| Pixel size(Å) | 0.82 |
| Symmetry imposed | D2 |
| Number of collected movies | 5387 |
| Initial particle images (no.) | 989280 |
| Final particle images (no.) | 95054 |
| Map resolution (Å) | 3.3 |
| FSC threshold | 0.143 |
| Map resolution range (Å) | 3.2-4.3 |
| Refinement |  |
| Initial model used (PDB code) | Alphafold |
| Map sharpening B-factor(Å2) | -120 |
| Model composition |  |
| Non-hydrogen atoms | 17456 |
| Protein residues | 2164 |
| Ligands | CTP |
| Water | 0 |
| Ions | 8 |
| B factors(Å2) |  |
| Protein | 54 |
| Ligand | 25 |
| Water | - |
| R.m.s. deviations |  |
| Bond lengths (Å) | 0.00 |
| Bond angles (°) | 0.69 |
| Validation |  |
| MolProbity score | 1.39 |
| Clashscore | 7.01 |
| Poor rotamers (%) | 0.00 |
| Ramachandran plot |  |
| Favored (%) | 98.04 |
| Allowed (%) | 1.96 |
| Disallowed (%) | 0.00 |


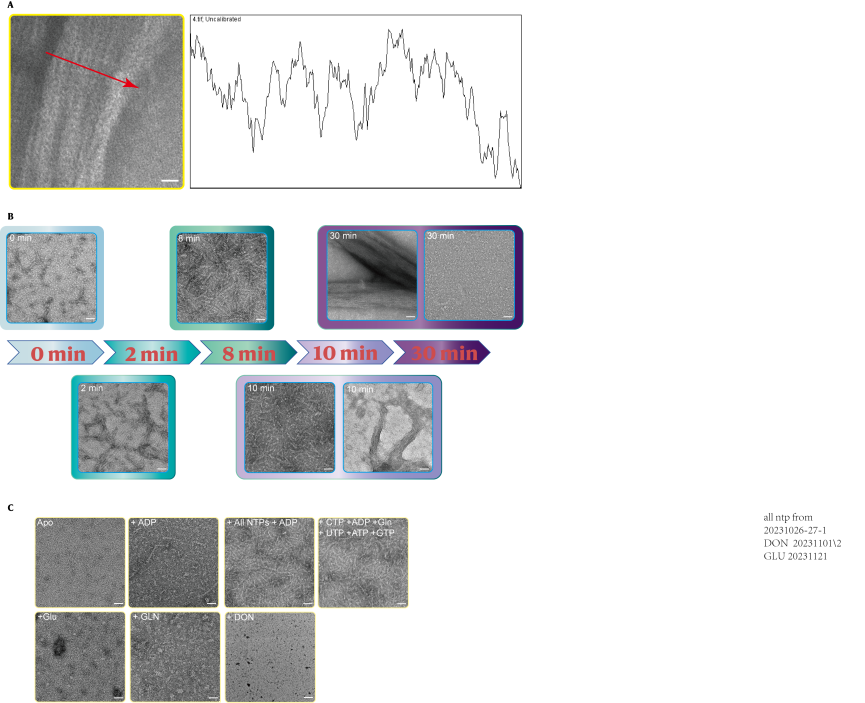


**Figure S1. Grayscale analysis of bundle structure, time experiment of hCTPS1 combined with CTP and negative staining of human hCTPS1 under several other states.**

1. The grayscale map shows the density information of the bundle structure from left to right for the area pointed by the red arrow. The scale bar of the negative-staining EM micrographa is 200 nm.
2. Negative-staining EM micrographs of hCTPS1 binding with CTP at 0, 2,8 10, 30 min. The two pictures in the same solid boxes (at 10, 30 min) are the results of different positions on the same grid. The scale bar is 50 nm.
3. Negative staining of human hCTPS1 in: Apo, +ADP, +all NTPs and ADP, + all ligands, +Glu, +GLN, +DON state. The scale bar is 50 nm.

_
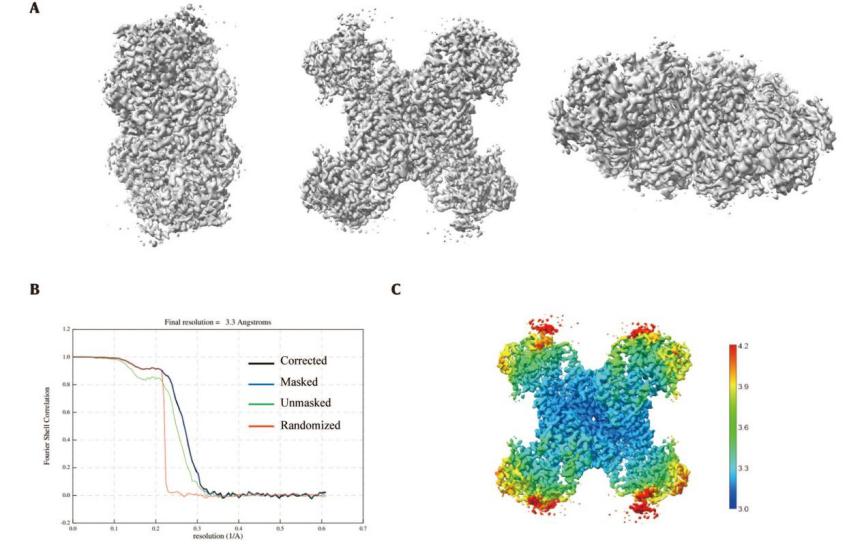
_

**Figure S2. Statistics of the final density maps of the hCTPS1 p-state tetramer.**

1. Three views of the final tetramer model.
2. Gold-standard FSC curve of the final density maps of the hCTPS1 bound with CTP model.
3. Local resolution map of the hCTPS1 p-state tetramer 3D refinement density map.


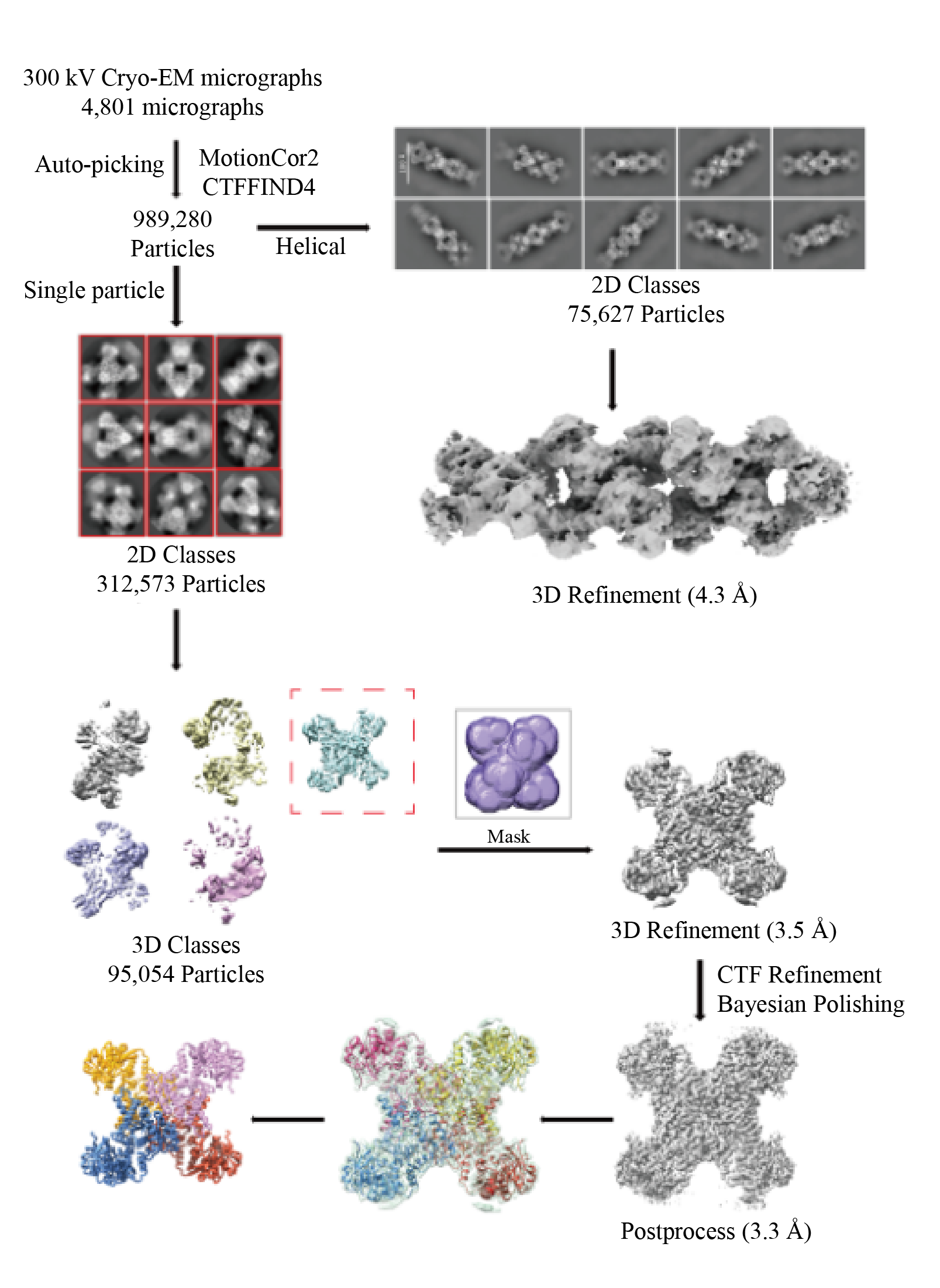


**Figure S3. Image processing work flow of CTP-binding hCTPS1 tetramer.**

We collected 989,280 particles from 4,801 micrographs while kept 312,573 particles for single particle refinement and 75,627 particles for helical refinement after 2D classification. After 3D classification, 3D refinement and correction, final map and model was generated through different reconstructions.


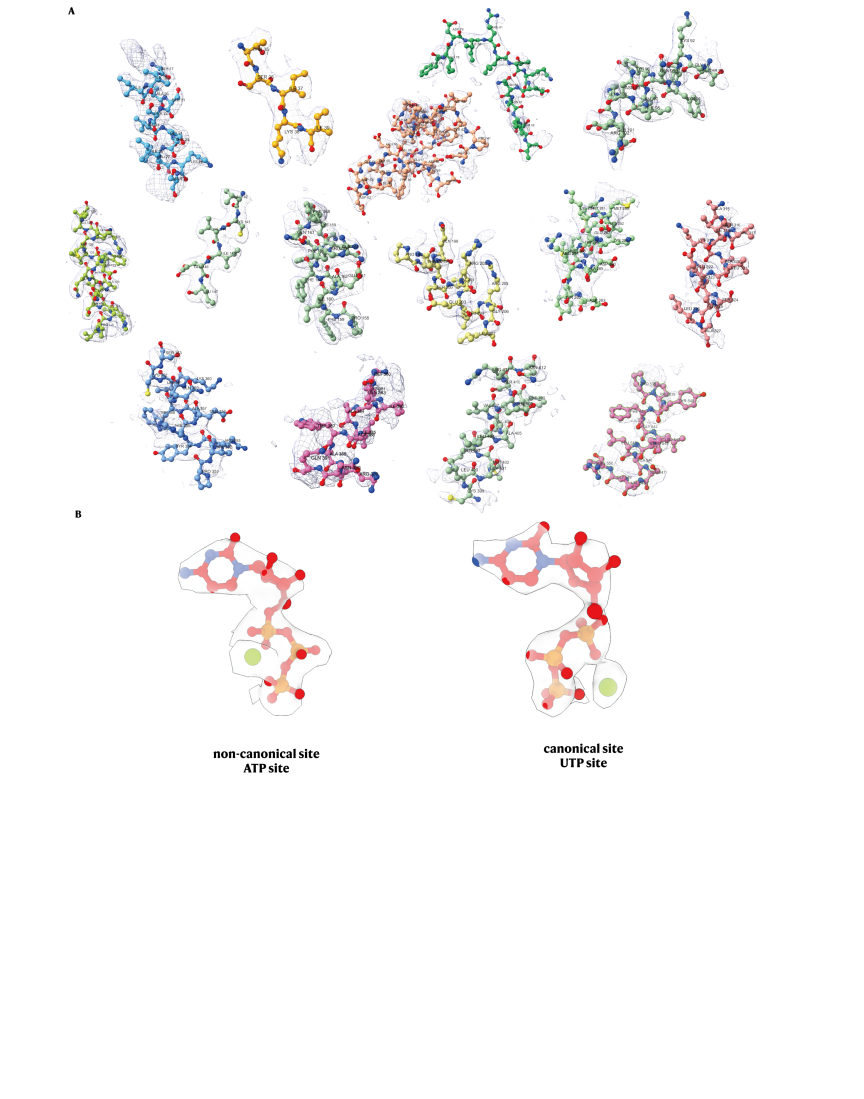


**Figure S4. Representative electron density map of individual regions of hCTPS1 model.**

(A) The electron density map of representative secondary structure of hCTPS1.

(B) The electron density map of two bound CTPs.


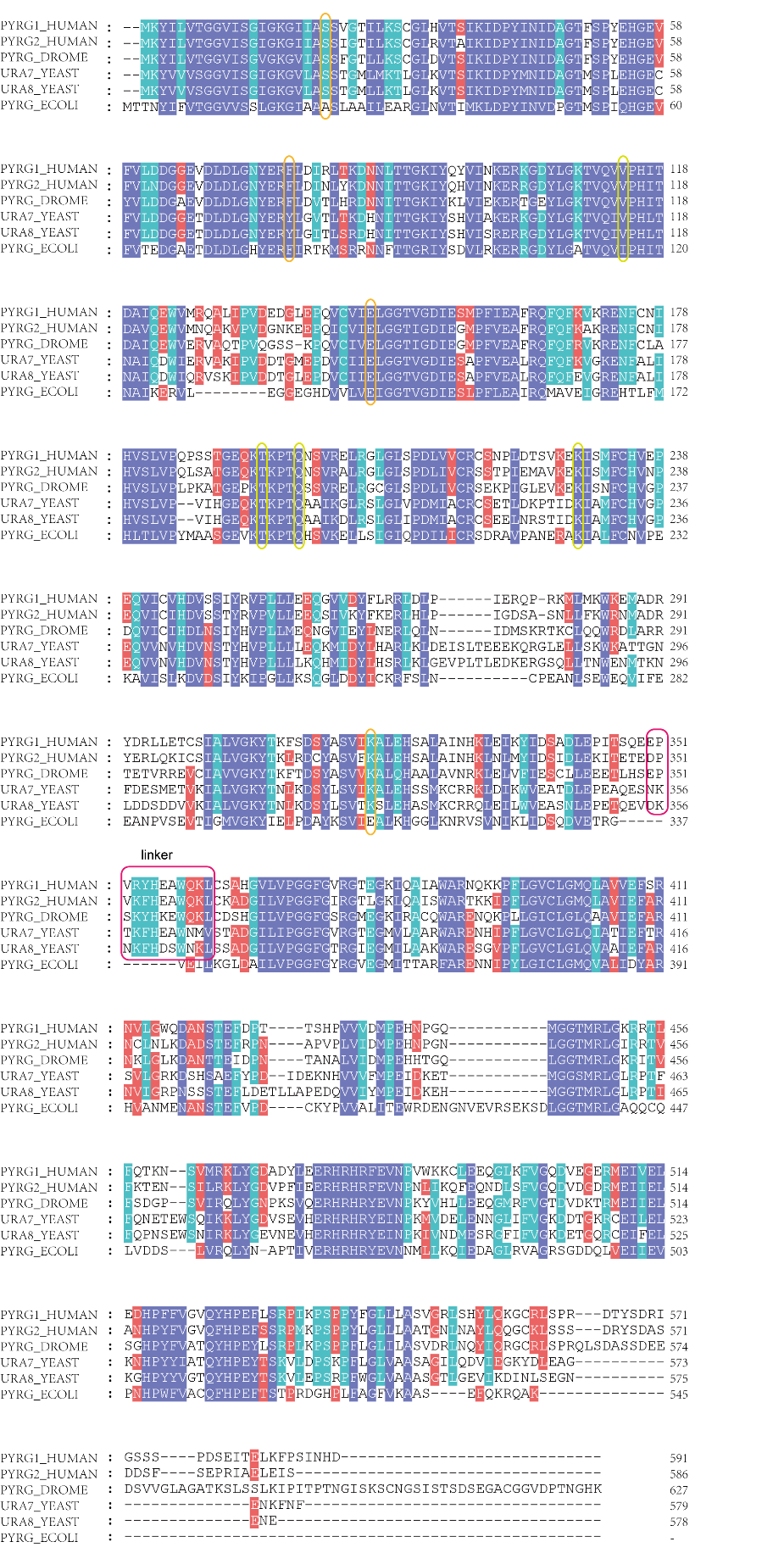


**Figure S5. Align of CTPS sequences of various species.**

The boxes marked by the red represent the residue between the two domains where linker is located. The yellow boxes marked the residues involved in identification of the canonical binding pocket and the orange ones marked key residues in the non-canonical binding pocket.


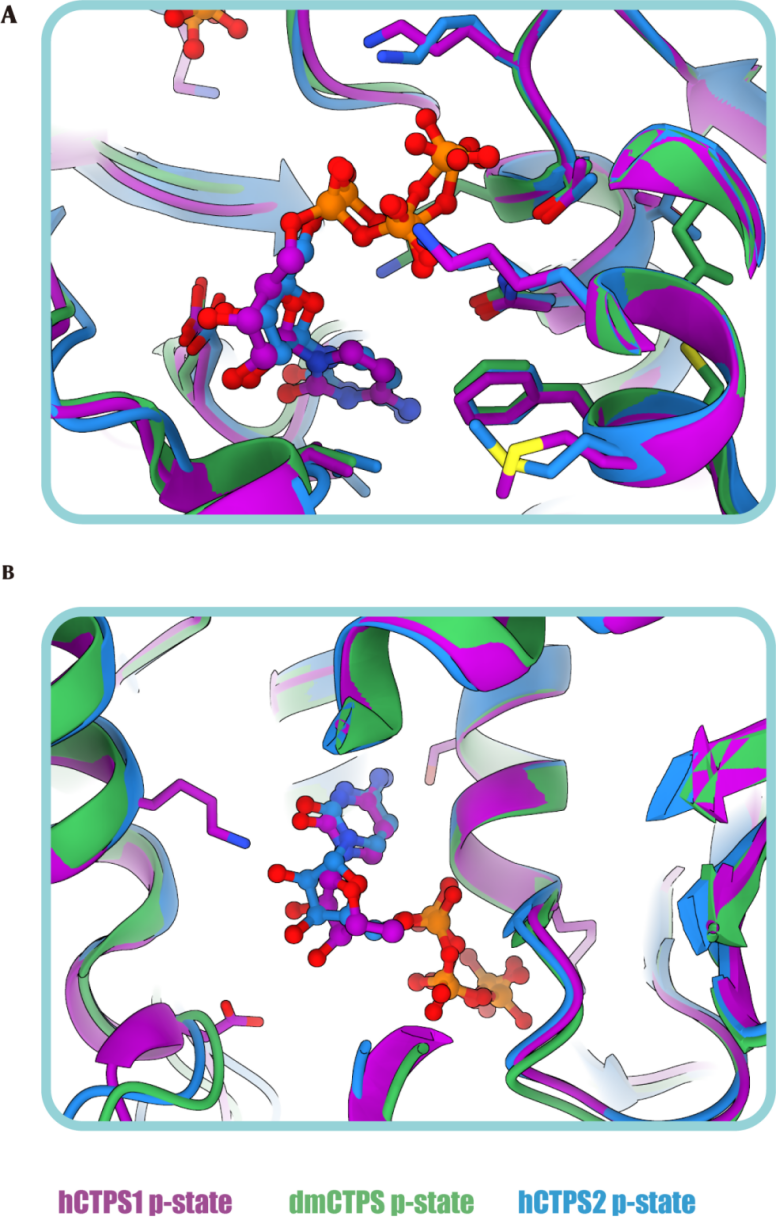


**Figure S6. The comparison of hCTPS1, hCTPS2 and DmCTPS at two CTP binding pockets.**

The hCTPS1 p-state model is shown in purple, the dm-CTPS p-state model is shown in forest green and the hCTPS2 p-state model is shown in deep sky blue.

1. Canonical binding pocket
2. Non-canonical binding pocket


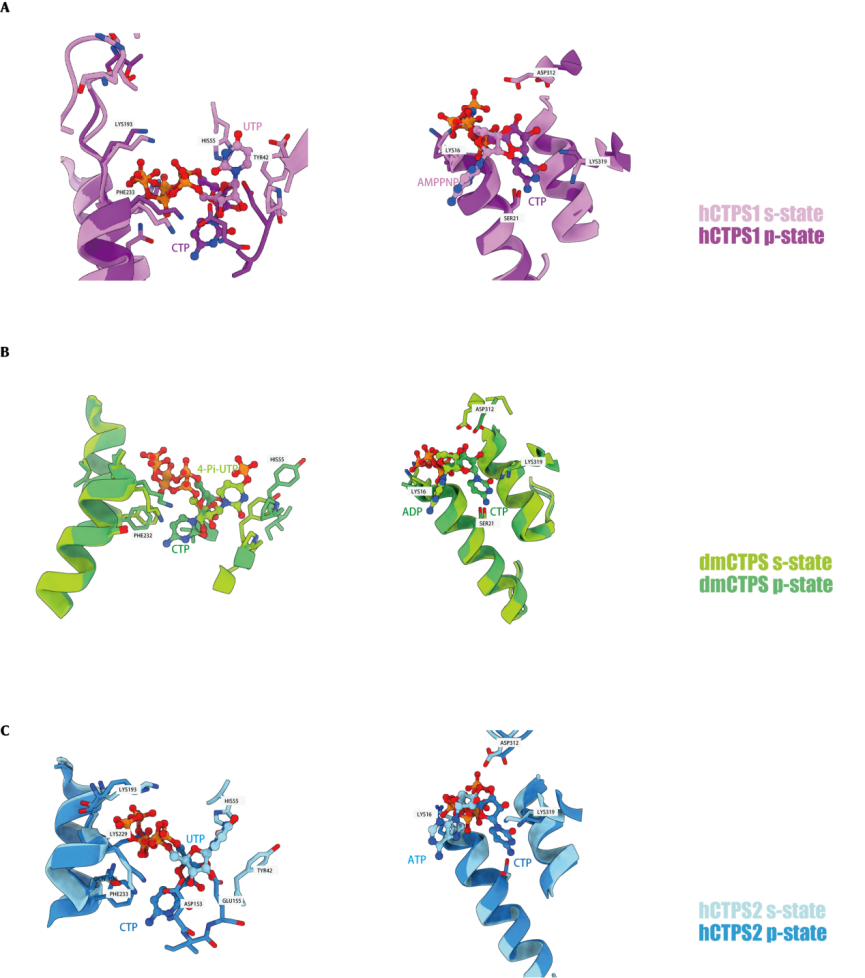


**Figure S7. The comparison of hCTPS1, DmCTPS and hCTPS2 under substrate and product conditions.**

The structures of hCTPS1 (7mgz, bound with UTP and AMPPNP), DmCTPS (7dpt, bound with 4-Pi-UTP and ADP) and hCTPS2(6pk4, bound with UTP and ATP) and DmCTPS in s-state are colored in pink, lime green and powder blue, respectively. The structural of hCTPS1(9vmm, bound with CTP), DmCTPS (7dpw, bound with CTP) and hCTPS2 (7mh1, bound with CTP) in p-state are colored in purple, forest green and deep sky blue, respectively.


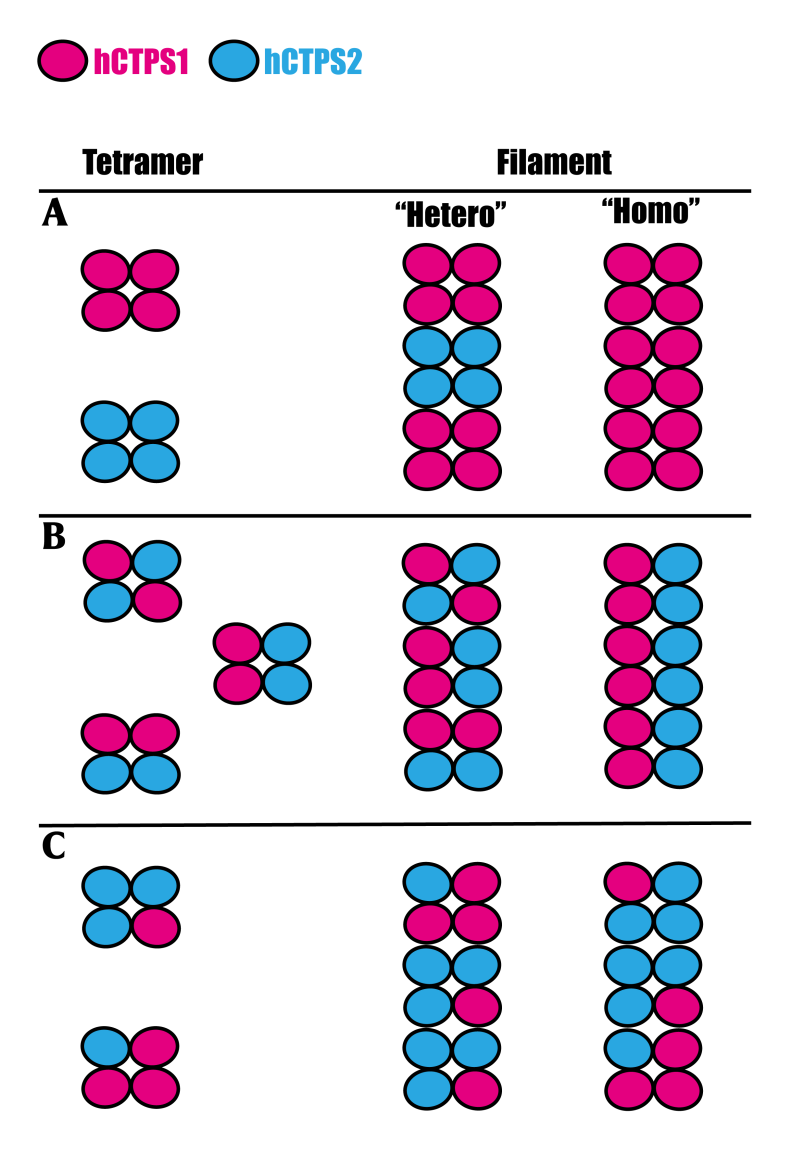


**Figure S8. Possible assemble mechanisms of hCTPS isoforms tetramer and filament.**

1. One or two homo-tetramers assemble into heterogeneous filament structures through analogous interaction interface.
2. Chimeric tetramers, each formed by 2 hCTPS1 monomers and 2 hCTPS2 monomers, with symmetrical structure, assemble into filament through binding to ligands.
3. Various hCTPS isoform monomers from heterogeneous tetramers and further assemble into irregular filament through similar interface.
